# Taxonomic and Functional Differences between Microbial Communities in Qinghai Lake and Its Input Streams

**DOI:** 10.3389/fmicb.2017.02319

**Published:** 2017-11-22

**Authors:** Ze Ren, Fang Wang, Xiaodong Qu, James J. Elser, Yang Liu, Limin Chu

**Affiliations:** ^1^Flathead Lake Biological Station, University of Montana, Polson, MT, United States; ^2^State Key Laboratory of Simulation and Regulation of Water Cycle in River Basin, China Institute of Water Resources and Hydropower Research, Beijing, China; ^3^Department of Water Resources, China Institute of Water Resources and Hydropower Research, Beijing, China; ^4^Department of Water Environment, China Institute of Water Resources and Hydropower Research, Beijing, China

**Keywords:** co-occurrence, functional, microbial community, stream-lake linkage, taxonomic

## Abstract

Understanding microbial communities in terms of taxon and function is essential to decipher the biogeochemical cycling in aquatic ecosystems. Lakes and their input streams are highly linked. However, the differences between microbial assemblages in streams and lakes are still unclear. In this study, we conducted an intensive field sampling of microbial communities from lake water and stream biofilms in the Qinghai Lake watershed, the largest lake in China. We determined bacterial communities using high-throughput 16S rRNA gene sequencing and predicted functional profiles using PICRUSt to determine the taxonomic and functional differences between microbial communities in stream biofilms and lake water. The results showed that stream biofilms and lake water harbored distinct microbial communities. The microbial communities were different taxonomically and functionally between stream and lake. Moreover, streams biofilms had a microbial network with higher connectivity and modularity than lake water. Functional beta diversity was strongly correlated with taxonomic beta diversity in both the stream and lake microbial communities. Lake microbial assemblages displayed greater predicted metabolic potentials of many metabolism pathways while the microbial assemblages in stream biofilms were more abundant in xenobiotic biodegradation and metabolism and lipid metabolism. Furthermore, lake microbial assemblages had stronger predicted metabolic potentials in amino acid metabolism, carbon fixation, and photosynthesis while stream microbial assemblages were higher in carbohydrate metabolism, oxidative phosphorylation, and nitrogen metabolism. This study adds to our knowledge of stream-lake linkages from the functional and taxonomic composition of microbial assemblages.

## Introduction

Microbial communities are fundamental components in aquatic environments and play a crucial role in driving global energy fluxes and biogeochemical cycling (Falkowski et al., 2008). Bacteria, for example, strongly influence carbon, nitrogen, phosphorus, and sulfur fluxes in marine, lacustrine, and fluvial ecosystems (Battin et al., 2003; DeLong et al., 2006; Danger et al., 2007; Fuhrman, 2009; Peter and Sommaruga, 2016). Microorganisms encompass tremendous diversity (Lennon and Locey, 2016) and have immense cumulative biomass and activities (Whitman et al., 1998). Thus, understanding the taxonomic and functional compositions of microbial communities is of great interest and importance because it may shed light on ecosystem processes and community assembly mechanisms (Van der Gucht et al., 2007; Hanson et al., 2012).

In stream ecosystems, a majority of microorganisms occur in benthic biofilms (Findlay, 2010), where they play a key role in biogeochemical cycling and are responsible for organic matter (OM) mineralization, nutrient uptake, the transfer of nutrients to higher trophic levels, as well as immobilization and transformation of contaminants (Schiller et al., 2007; Buchkowski et al., 2015). In stream biofilm assemblages, various heterotrophic and autotrophic taxa are tightly linked to each other via trophic and competitive interactions (Fitter and Hillebrand, 2009). In lake ecosystems, microbial communities have an extremely high level of genetic diversity and also play a key role in biogeochemical cycles (Newton et al., 2011; Hayden and Beman, 2016; Huang et al., 2016). Thus, taxonomic and functional changes of microbial communities in stream biofilms and lake water may contribute to changes in ecosystem processes (Ylla et al., 2014; Wilhelm et al., 2015; Zwirglmaier et al., 2015; Peter and Sommaruga, 2016).

In watersheds, lakes and their input streams are highly linked in multiple ways (Cole et al., 2006; Marcarelli and Wurtsbaugh, 2009; Jones, 2010; Ylla et al., 2013). Streams are the primary receiver of nutrients and OM inputs from terrestrial ecosystems (Vannote et al., 1980; Figueiredo et al., 2010; Deegan et al., 2011). Lakes have an intimate relationship with catchment characteristics through material transport by surface runoff from their input streams and rivers (Cole et al., 2006; Zhang, 2011; Canham et al., 2012; Cronan, 2012; Sadro et al., 2012). It has long been of great interest to explore the relationships between stream properties and downstream aquatic ecosystems. However, the relationships between microbial communities in lakes and their input streams are not well-understood, limiting our understanding of ecosystem structures and functions and hindering effective management and protection of aquatic ecosystems.

Different biomes typically harbor distinct microbial assemblages (Fierer et al., 2012; Hugerth et al., 2015; Louca et al., 2016). However, it has been suggested that closely related taxa may have very different functional attributes and distinct taxa can share specific functional traits (Allison and Martiny, 2008; Philippot et al., 2010; Fierer et al., 2012; Dopheide et al., 2015). Thus, elucidating taxonomic and functional differences of microbial assemblages in streams and lakes is important to get insights into their roles in the ecosystem processes they promote (Freedman and Zak, 2015; Wang et al., 2016) and understand biogeochemical cycles in lakes and streams as well as lake-stream linkages (Louca et al., 2016). Moreover, aquatic ecosystems are facing increasing pressures from various anthropogenic impacts in their watersheds (Abell et al., 2011; Erol and Randhir, 2013; Umbanhowar et al., 2015), influencing community composition, biomass, as well as functions in both streams and lakes (Fanta et al., 2010; Hill et al., 2011; Drake et al., 2012). Thus, revealing the relationships between stream and lake microbial assemblages is also important for understanding how the threats from watershed are transferred to adjacent streams and to further downstream aquatic ecosystems.

Taxonomic and functional differences of microbial communities in lake water and stream biofilms still remains unclear (Crump et al., 2007; Ylla et al., 2013; Zwirglmaier et al., 2015). In this study, we used Qinghai Lake and its input streams as an example. Qinghai Lake is the largest lake in China located on Qinghai-Tibet Plateau maintaining ecological security of northeast Qinghai-Tibet Plateau. Terrestrial and aquatic ecosystems in the Qinghai Lake watershed are sensitive to global climate change and other anthropogenic impacts (An et al., 2006; Hao, 2008; Li et al., 2009). We conducted an intensive field sampling of microbial communities from lake water and stream biofilms in this area. We determined bacterial communities using high-throughput 16S rRNA gene sequencing and predicted functional profiles using PICRUSt (Phylogenetic Investigation of Communities by Reconstruction of Unobserved States) to determine the taxonomic and functional differences between microbial communities in stream biofilms and lake water. We tried to address these basic questions: do stream biofilms harbor microbial communities that are taxonomically distinct from those found in lake water? What functional attributes distinguish or connect microbial communities in stream biofilms and lake water?

## Materials and Methods

### Study Area

**FIGURE 1:**
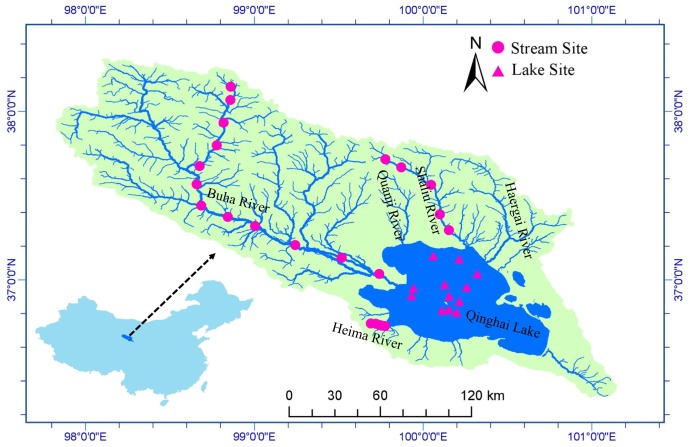
Study area and sample sites. Qinghai Lake is the largest lake in China and located on Qinghai-Tibet Plateau. Water samples and microbial samples were collected at 22 stream sites and 12 lake sites. The map was created in ArcGIS 14.0 (http://desktop.arcgis.com/en/arcmap/) using DEM image download from USGS (https://earthexplorer.usgs.gov/).

Qinghai Lake (QL, 36°32′–37°15′ N, 99°36′–100°47′ E) is a remote endorheic saline lake located at 3194 m above sea level on the Qinghai-Tibet Plateau (**Figure 1**). The lake lies at a junction of three major climatic systems, the Westerlies, the East Asian, and Indian summer monsoons (An et al., 2012; Chen et al., 2015). It is the largest lake in China with a surface area of 4260 km^2^, a catchment area of 29,660 km^2^, and an average depth of 21 m (Li et al., 2007). Mean annual precipitation in the basin is 389.1 mm and average annual evaporation is 895.4 mm. Annual mean temperature is -0.3°C with a linear warming rate of 0.28°C/10a (Chen et al., 2011). There are more than 40 rivers and streams flowing into Qinghai Lake but most of them are seasonal. There are five main tributaries: Buha River, Shaliu River, Haergai River, Quanji River, and Heima River (**Figure 1**). Together, these tributaries contribute 83% of the total runoff (Li et al., 2007).

Qinghai Lake area has terrestrial and aquatic ecosystems that are sensitive to global climate change and other anthropogenic impacts (An et al., 2006; Hao, 2008; Li et al., 2009). Grassland is the main landcover type, accounting for 75% area of the watershed. However, the grassland is seriously deteriorating due to overgrazing and climate change, with degraded grassland accounting for 37% area of the whole watershed (Luo et al., 2013). Grassland degradation influences biogeochemical processes and ecosystem stability in both terrestrial and aquatic habitats (Scott et al., 2001; Chen et al., 2013).

### Field Sampling

We collected samples from 22 stream sites (12 in Buha River, 5 in Shaliu River, and 5 in Heima River) and 12 lake sites during June 23 and 29, 2016 (**Figure 1**). Water samples were collected for chemical analyses in the laboratory. At each stream sampling point, 6 to 9 submerged rocks were randomly chosen along the river cross section. The benthic biofilm was removed by rigorously brushing a 4.5-cm-diameter area from the upper surface of each stone with a sterilized nylon brush (changed between samples) and rinsing the slurry with sterile water. Approximately, 10 mL of the mixed slurry was filtered through 0.2-μm membrane filters that were immediately frozen in liquid N in the field. At each lake sample site, surface water samples for microbial analyses were collected at a depth of 0.5 m. 600 ml water were filtered onto 0.2-μm membrane filters that were immediately frozen in liquid nitrogen in the field. When transported to the lab, the microbial samples were stored at -80°C until DNA extraction. Water samples were acid fixed and transported to the laboratory at 4°C.

### Physicochemical Parameters

At each sample site, water temperature (Temp), dissolved oxygen (DO), pH, and conductivity (Cond) were measured *in situ* using a YSI handheld meter (model 80; YSI, Yellow Springs, OH, United States). Elevation was measured using a GPS unit (Triton 500, Magellan, Santa Clara, CA, United States). Water samples were collected for nutrients and dissolved organic carbon (DOC) analyses. Total nitrogen (TN) was quantified by ion chromatography after persulfate oxidation (EPA 300.0). Nitrate (NO_3_^-^) was determined by ion chromatography (EPA 300.0). Ammonium (NH_4_^-^) was determined using the indophenol colorimetric method (EPA 350.1). Total phosphorus (TP) was analyzed using the ascorbate acid colorimetric method after oxidation (EPA 365.3). Soluble reactive phosphorus (SRP) was quantified using the ascorbate acid colorimetric (EPA 365.3). DOC was analyzed using a Shimadzu TOC Analyzer (TOC-VCPH, Shimadzu Scientific Instruments, Columbia, MD, United States). The physicochemical parameters were shown in Supplementary Table S1.

### DNA Extraction, PCR, and Sequencing

Bacterial 16S rRNA genes were analyzed to determine the benthic biofilm and pelagic community structure and diversity. Genomic DNA was extracted using the PowerSoil DNA Isolation Kit (MoBio, Carlsbad, CA, United States) following manufacturer protocols. The V3–V4 regions of the 16S rRNA gene were amplified using 338F-ACTCCTACGGGAGGCAGCA and 806R-GGACTACHVGGGTWTCTAAT (Invitrogen, Vienna, Austria). PCR was performed with a model 2720 thermal cycler (ABI, United States) using the following program: 1-min hot start at 80°C, 94°C for 5 min followed by 30 cycles of denaturation at 94°C for 30 s, followed by annealing at 52°C for 30 s, at 72°C for 1 min 30 s, with a final extension step at 72°C for 10 min. Amplified DNA was verified by electrophoresis of PCR mixtures in 1.0% agarose in 1X TAE buffer and purified using the Gel Extraction Kit (Qiagen, Hilden, Germany). Samples were sent for sequencing on a Miseq sequencing platform (Illumina, San Diego, CA, United States).

### Sequence Analysis and Functional Gene Prediction

Raw sequence data (available at National Center for Biotechnology Information, SRP 115613) were processed using the software package QIIME 1.9.0 (Caporaso et al., 2010). The forward and reverse reads were merged and assigned to samples based on barcode and truncated by cutting off the barcode and primer sequence. Quality filtering on merged sequences was performed and sequences which did not meet the following criteria were discarded: sequence length < 200 bp, no ambiguous bases, and mean quality score ≥ 20. Then the sequences were compared with the reference database (RDP Gold database) using UCHIME algorithm (Edgar et al., 2011) to detect chimeric sequence, and then the chimeric sequences were removed. The effective sequences were grouped into operational taxonomic units (OTUs) using the clustering program VSEARCH 1.9.6 (Edgar, 2010) against the Silva 123 database pre-clustered at 97% sequence identity level. The Ribosomal Database Program (RDP) classifier was used to assign the taxonomic category to all OTUs at a confidence threshold of 0.8. The RDP classifier uses the Silva 123 database which has taxonomic categories predicted to the species level.

Functional potential of bacteria communities in the stream biofilm and lake water samples were predicted using PICRUSt 1.1.0 (Langille et al., 2013). PICRUSt is a bioinformatics tool that predicts the functional composition of a metagenome using 16S rRNA sequences and a reference genome database. Using an extended ancestral-state reconstruction algorithm, PICRUSt predicts which gene families are present and then combines gene families to estimate the composite metagenome (Langille et al., 2013). Sequences used for PICRUSt prediction were clustered into OTUs (97% similarity) against the Greengenes 13.5 database using QIIME 1.9.0. Then the rarefied OTU table was used for predicted 16S rRNA gene copy number normalization. The normalized-OUT table was used to predict the functional genes, and the accuracy of the metagenome prediction was assessed by the nearest sequenced taxon index. Finally, the metagenome prediction was further classified into Kyoto Encyclopedia of Genes and Genomes (KEGG) Orthologs (KOs) at different pathway levels (levels 1–3).

### Analysis

We compared taxonomic and functional profiling of the bacteria communities to elucidate the differences and linkages between microbial assemblages in stream biofilm and lake water. To determine whether physicochemical factors, relative abundances of phyla, taxonomic and functional alpha diversities, and relative abundances of functional gene categories were significantly different between stream samples and lake samples, we conducted a bootstrap *t*-test using SPSS 20.0 (IBM, Armonk, NY, United States). Analysis of similarities (ANOSIM) was used to test whether sample categories harbored significantly different microbial communities or metagenomes (using PAST 3.0). Mantel tests were run to assess correlations between functional and taxonomic community dissimilarity matrices based on Bray–Curtis distance. Heatmaps were generated using Heatplus package (version 2.20.0) and Gplots package (version 3.0.1) in R (version 3.3.2) to reveal taxonomic and functional differences between communities based on Bray–Curtis distance. The non-metric multidimensional scaling (NMDS) was applied to reveal differences in community composition between lake and stream microbial assemblages (using R 3.3.2 and Vegan package 2.4-1).

Understanding microbial interactions is essential to reveal community assembly rules (Gotelli and McCabe, 2002; Fuhrman, 2009). For example, co-occurrence patterns can show how particular organisms in a system occur together and vary in a changing world (Fuhrman, 2009), and the direct and indirect interactions may help to ascertain the functional roles or ecological niches occupied by microorganisms (Fuhrman and Steele, 2008; Chaffron et al., 2010). The relative abundances of the OTUs in each sample were used to construct matrices for visualizing interactions between OTUs in networks (stream microbial network and lake microbial network). A Spearman correlation coefficient R score and a *P*-value were calculated pairwise between OTUs (for OTUs with relative abundance higher than 0.01%) using the Hmisc package (version 4.0-1) in R (version 3.3.2). Only strong (Spearman’s correlation coefficient *R* > 0.70 or *R* < -0.70) and significant (*P* < 0.01) correlations were considered. These correlations were visualized using Cytoscape (version 3.4.0). Each node represents an OTU, and each edge represents a strong and significant correlation. To describe the network topology, a set of node/edge metrics (Supplementary Table S3) were analyzed using the Network Analyzer plugin within Cytoscape (Assenov et al., 2008). The modular structure analysis of each network was conducted using the ClusterMaker app in Cytoscape. The modularity was calculated (Su et al., 2010), and the modularity value > 0.4 suggests that the network is modular (Newman, 2006). Since only a single data point was available for each network topological parameter of each real network (stream microbial network and lake microbial network), standard statistical analysis could not be performed to assess their statistical significance (Zhou et al., 2010). Thus, referring to the method proposed by Zhou et al. (2010), the random network construction and network comparison were conducted. For each real network (stream microbial network and lake microbial network), a total of 100 random networks with the same size as the real network were generated using the Network Randomizer app (version 1.1.2) and all of the network topological parameters were calculated individually (Zhou et al., 2010; Kuang et al., 2017). Then, the statistical *Z*-test was employed to test the differences between the topological parameters of the real network and its random networks, using the average and standard deviation for each parameter of all of the random networks. Meanwhile, the Student’s *t*-test was used to compare the two real networks (stream microbial network and lake microbial network) using the standard deviations derived from corresponding random networks (Zhou et al., 2010; Kuang et al., 2017).

## Results and Discussion

### General Comparison of Community Composition

**FIGURE 2:**
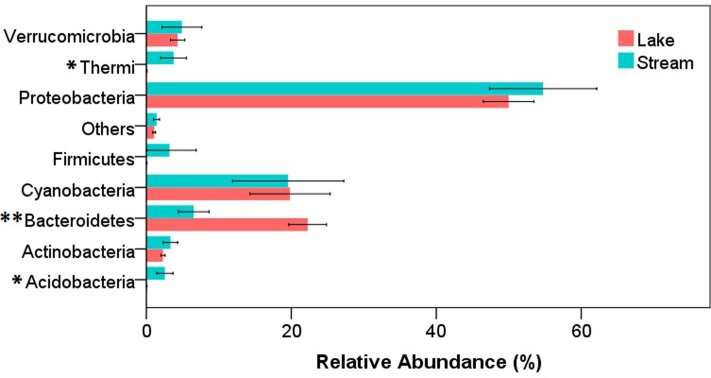
Relative abundance of microorganisms at phylum level in stream biofilms and lake water. Only the phyla that had a relative abundance > 1% either in stream or lake were shown. “Others” represent the unsigned OTUs and the phyla with a relative abundance < 1%. ^∗^*P* < 0.05, ^∗∗^*P* < 0.01.

After quality filtering, a total of 343,350 reads were obtained from the 34 samples (12 lake samples and 22 stream samples). In both stream and lake microbial communities, the dominant phyla were Proteobacteria, Bacteroidetes, and Cyanobacteria. At phylum level, there were significant differences between lake and stream in the relative abundance of Acidobacteria (*P* < 0.05), Bacteroidetes (*P* < 0.01), and Thermi (*P* < 0.05) (**Figure 2**). Lake water had a higher relative abundance of Bacteroidetes but a lower relative abundance of Acidobacteria and Thermi than stream biofilms. Previous research about the microbial communities in Qinghai Lake mainly focused on lake itself, including the microbial communities in lake water column (Huang et al., 2014), water-sediment interface (Dong et al., 2006), and sediment (Jiang et al., 2009). It was revealed that autotrophic Cyanobacteria and heterotrophic Proteobacteria dominated the DNA and RNA samples, respectively (Huang et al., 2014), and the proportion of the Proteobacteria decrease from the bottom of the lake to the sediment (Dong et al., 2006). However, our research was the first to compare the microbial communities in Qinghai Lake and its input streams.

Functional alpha diversity, the alpha diversity of protein-coding gene categories identified within a metagenome, can provide important information for the diversity and distributions of functional traits or functional genes across communities (Petchey and Gaston, 2002; Green et al., 2008; Gilbert et al., 2010; Fierer et al., 2012). In our study, both the taxonomic alpha diversity (the alpha diversity of taxa contained within an individual community) and the functional alpha diversity were significantly higher (*t*-test, *P* < 0.05) in stream than in lake samples (Supplementary Table S2), suggesting that streams have more niche range that can harbor more diverse microorganisms than the water column of the lake.

### Community Differences – Taxonomic and Functional

**FIGURE 3:**
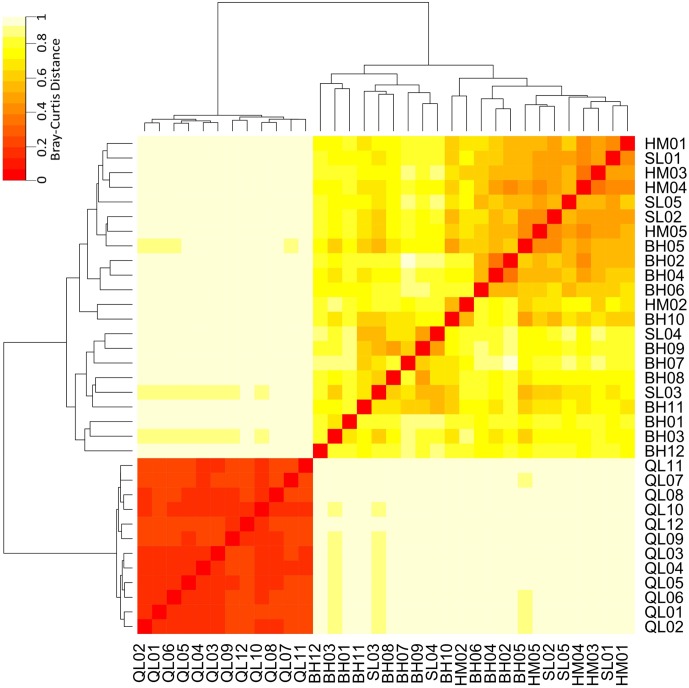
Heatmap showing the taxonomic differences of microbial communities between streams and Qinghai Lake based on Bray–Curtis distance. Bray–Curtis distances were calculated using relative abundances of OTUs.

**FIGURE 4:**
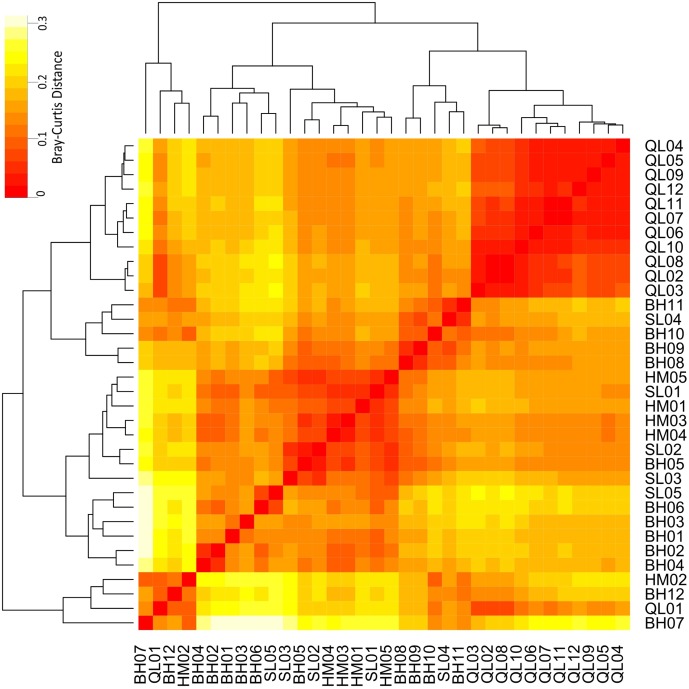
Heatmap showing the functional differences of microbial communities between streams and Qinghai Lake based on Bray–Curtis distance. Bray–Curtis distances were calculated using relative abundances of PICRUSt predicted KEGG orthologies (KOs).

Taxonomic differences between the microbial communities in stream biofilms and lake water were evident (**Figure 3**). Heatmap (**Figure 3**) showed that the communities in lake water were clustered apart from the stream biofilm communities. ANOSIM analysis also showed the distinction (*r* = 0.99, *P* < 0.001). Moreover, the communities in lake were more taxonomically similar to each other (low Bray–Curtis distance, **Figure 3**) than were stream communities to each other (high Bray–Curtis distance, **Figure 3**). Based on PICRUSt predicted KEGG orthologies (KOs), the heatmap (**Figure 4**) showed that the communities in lake were more functionally similar to each other than were the stream communities. However, the functional differences between microbial communities in stream biofilms and lake water were not significant (ANOSIM *r* = 0.13, *P* < 0.05) compared to taxonomic differences.

### Bacteria Co-occurrence

**FIGURE 5:**
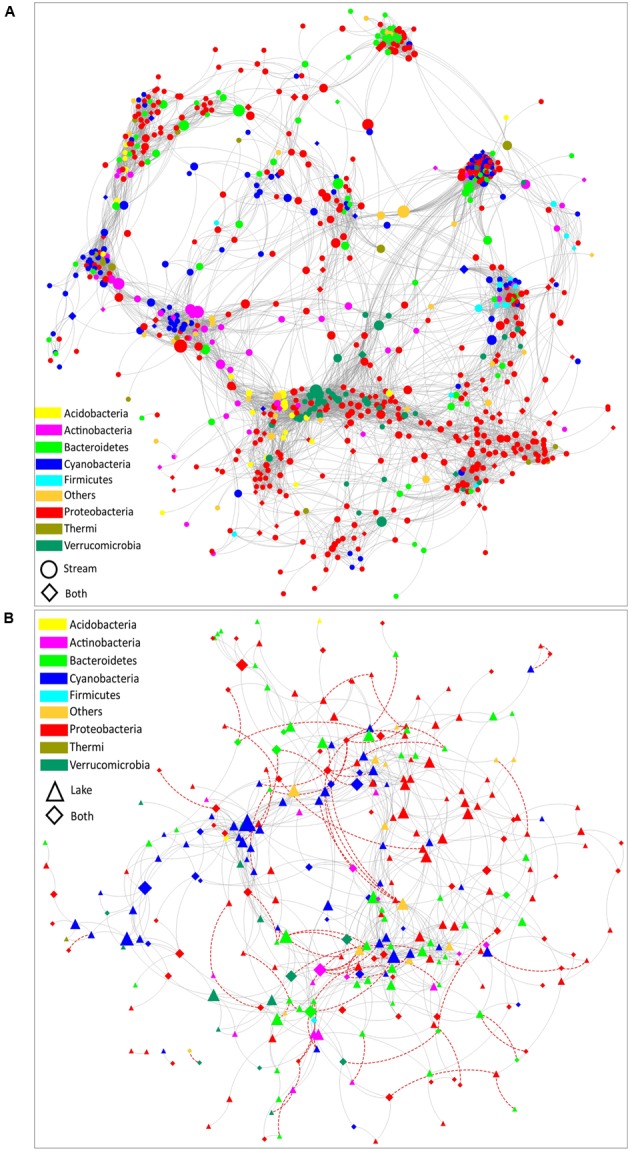
The co-occurrence network of bacterial communities in **(A)** stream biofilms and **(B)** lake water. Edges represent correlation relationships. Gray solid lines indicate positive associations (co-occurrence interactions) and red dashed lines indicate negative associations (co-exclusion interactions). Only strong and significant relationships (spearman *R* > 0.7 or *R* < –0.7, *P* < 0.01) are shown. Nodes are sized by the OTU betweenness and colored by phylum. Circle nodes represent OTUs with a relative abundance higher than 0.01% only in the stream samples. Triangle nodes represent the OTUs with a relative abundance higher than 0.01% only in the lake samples. Diamond nodes represent the OTUs with a relative abundance greater than 0.01% in both lake and stream samples.

Co- occurrence patterns of organisms have been evaluated to reveal community assembly rules and interaction networks in highly complex systems (Gotelli and McCabe, 2002; Fuhrman, 2009). In order to compare the co-occurrence networks between microbial communities in stream and lake samples, a stream microbial network (**Figure 5A**) and a lake microbial network (**Figure 5B**) were built. Several topological parameters were calculated to describe the interactions between OTUs (Supplementary Table S3). These structural properties permit exploration of how habitat traits are associated with the assembly of microbial communities (Barberan et al., 2012; Freedman and Zak, 2015). Overall, the stream microbial network contained 808 nodes (i.e., OTUs) with 10,542 edges (i.e., significant interactions). The lake microbial network was much less complex, containing 302 nodes with 895 edges. All the correlations were positive in the stream microbial network, while in lake microbial network, 8.3% of the correlations were negative (**Figure 5**), indicating that lake microorganisms have more competing relationships than stream microorganisms because of the homogeneous habitat and limited resources in the lake compared to the streams.

Both networks had a strongly clustered topology. Compared to the topology of randomly generated networks with the same size (Supplementary Table S3), the real networks exhibited a higher network centralization and clustering coefficient. Moreover, the stream microbial assemblages had a more correlated and complex bacterial network topology than the lake assemblages. Comparing the topological parameters of these two networks (Supplementary Table S3), the stream microbial network exhibited a greater number of nodes, network centralization, network density, network heterogeneity, characteristic path length, average number of neighbors, and clustering centralization (Supplementary Table S3). This is of interest because network topology (the nodes distribution and interaction) can affect the stability of the system (Barberan et al., 2012; Freedman and Zak, 2015). In previous macroecology studies, communities with tightly connected species were shown to be more susceptible to disturbance (Montoya et al., 2006; Saavedra et al., 2011). The highly heterogenous nature of streams vs. the highly homogeneous nature of lake pelagic zones might have resulted in the different topological structures of the two networks we document here. Highly connected microbial networks in streams suggest that stream microbial communities are more vulnerable and sensitive to various disturbances.

The bacterial assemblages in both stream and lake habitats exhibited a modular structure (Supplementary Table S3, modularity values > 0.4 suggest that the network is modular, Newman, 2006). Modularity is a characteristic of large complex systems (Barabasi and Oltvai, 2004; Newman, 2006; Olesen et al., 2007). In a biotic network, highly interconnected species are grouped into a module, within which species interactions are more frequent and intensive than with the rest of the community (Newman, 2006; Freedman and Zak, 2015). It has been proposed that higher modularity might reflect more pronounced niche differentiation (Freedman and Zak, 2015). In our study, the modularity of the stream microbial network was notably higher than lake microbial network (Supplementary Table S3), which is consistent with the possibility that streams have more niche range (as suggested by the clustering topologies) and thus can offer more niches for organism.

### Functional Properties

**FIGURE 6:**
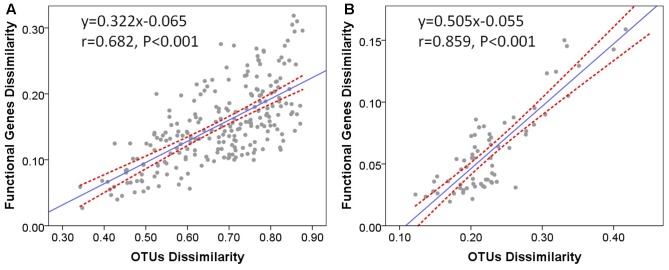
Bray–Curtis dissimilarities of functional genes versus Bray–Curtis dissimilarities of OTUs in **(A)** stream biofilms and **(B)** Qinghai Lake water. One point represents one sample pair. The Pearson correlation coefficient (r) and statistical significance (P) of linear regression are shown. Dotted lines indicate 95% confidence intervals.

It has already been demonstrated that functional beta diversity was strongly correlated with taxonomic and phylogenetic beta diversity across soil microbial communities (Fierer et al., 2012). To evaluate whether taxonomic differences between microbial communities are associated with their functional potential, we tested the relationships between functional and taxonomic composition at the community level. The Bray–Curtis dissimilarities between microbial communities were calculated from taxon abundances and functional gene abundances of microbial communities in stream biofilms and lake water, respectively. Mantel correlation tests revealed significant positive correlations between functional dissimilarities and taxonomic dissimilarities in stream biofilms (**Figure 6A**, *r* = 0.682, *P* < 0.001) and lake water (**Figure 6B**, *r* = 0.859, *P* < 0.001). Moreover, the relationship slope was smaller in stream than in lake. On the other hand, there were no significant correlations between physicochemical environment dissimilarities and functional dissimilarities (Mantel test, *P* > 0.05). These results suggest that, in stream biofilms and lake water, the overall functional differences between the microbial communities were significantly correlated with the differences of community composition. Moreover, different strains of a species may have distinct functions in lake water while there might be more different taxa which have the same functional traits in stream biofilms.

**FIGURE 7:**
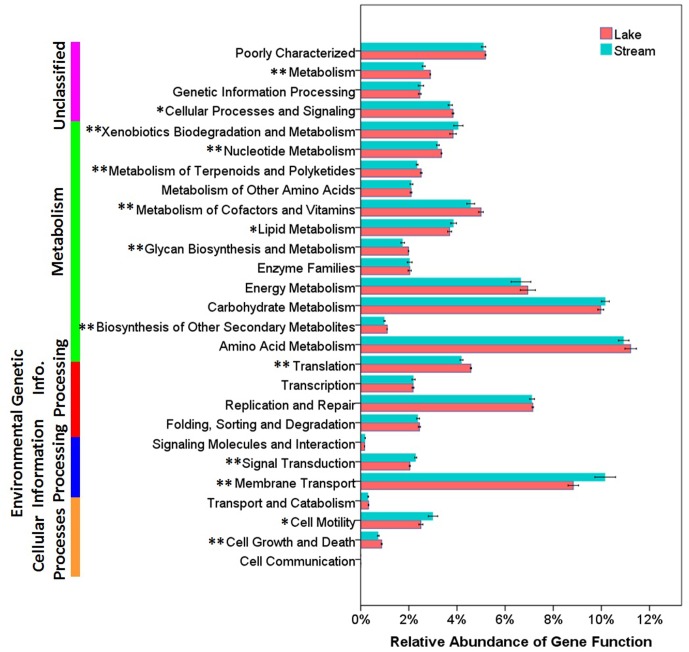
The relative abundance of various predicted functions of microbial communities in lake water and stream biofilms using PICRUSt grouped into level-2 functional categories. ^∗^ and ^∗∗^ indicate gene categories that are different between lake and stream at the *P* < 0.05 level and *P* < 0.01 significant level, respectively.

**FIGURE 8:**
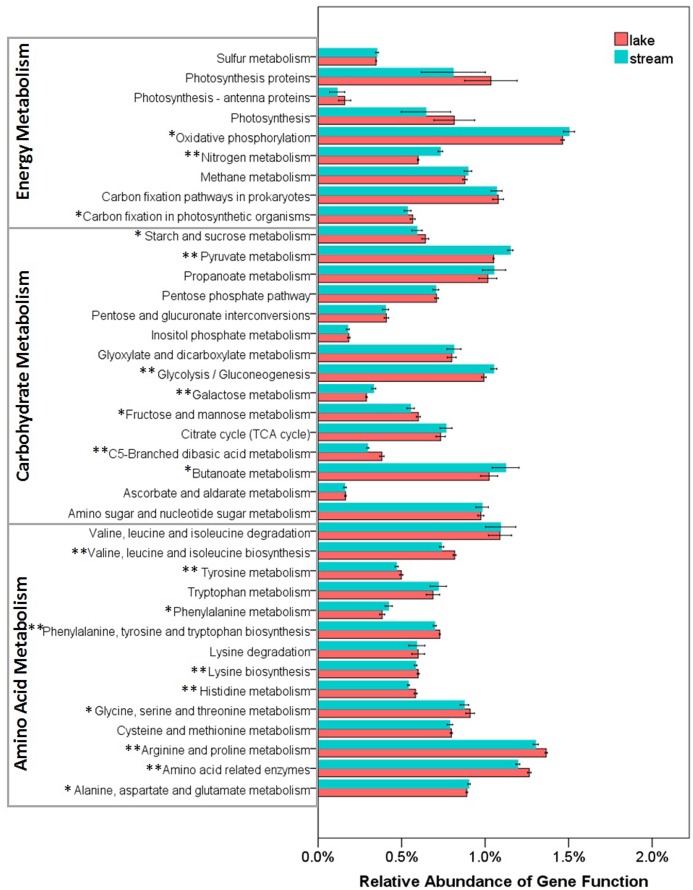
Relative abundance of various predicted functions of microbial communities in lake water and stream biofilms using PICRUSt grouped into level-3 functional categories. ^∗^ and ^∗∗^ indicate gene categories that are different between lake and stream at the *P* < 0.05 and *P* < 0.01 significant level, respectively.

Functional differences between lake and stream microbial communities were also evident from a comparison of the relative abundance of the PICRUSt predicted Kyoto Encyclopedia of Genes and Genomes (KEGG) orthologies (KOs) classified at level-1 (Supplementary Figure S1), level-2 (**Figure 7**), and level-3 (the lowest level of resolution, **Figure 8**). In microbial communities of both stream biofilms and lake water, the majority of KOs at level-1 involved metabolism pathways, followed by genetic information processing, unclassified, environmental information processing, cellular processes, and organismal systems (Supplementary Figure S1). These major gene categories were significantly different in abundance between stream and lake microbial communities (*t*-test, *P* < 0.0.5, Supplementary Figure S1). In general, microbial assemblages in lake water had higher relative abundance of genes associated with metabolism pathways and genetic information processing. The microbial assemblages in stream biofilms, however, had higher relative abundance of genes associated with environmental information processing and cellular processes.

In order to gain more insight into the functional differences, we compared the relative abundance of KOs at level-2. Microbial assemblages in lake water had higher relative abundance in many metabolism pathways, while the microbial assemblages in stream biofilms were characterized by genes associated with xenobiotic biodegradation and metabolism and lipid metabolism (*t*-test, *P* < 0.0.5, **Figure 7**). This is notable, as lipids is one of the major components of OM occurring in stream ecosystems (Thurman, 2012), and bacteria in benthic biofilms are the main decomposers of OM (Amon and Benner, 1996; Ylla et al., 2011). Moreover, streams are also the primary recipient of pollutants and xenobiotics inputs from the watershed (Tello et al., 2010; Lee et al., 2014). Consequently, most aquatic organisms, as well as bacteria, are exposed to these xenobiotics, which can be taken up, bioaccumulated, and degraded (van Leeuwen and Vermeire, 2007).

Several studies have reported that nutrient sources are potential drivers of microbial community structure and function in streams (Peter et al., 2011; Wilhelm et al., 2015) and in lakes (Judd et al., 2006). However, there were no significant differences between stream and lake microbial assemblages, in the relative abundance of three core resources metabolism pathways, carbohydrate metabolism, energy metabolism, and amino acid metabolism (**Figure 7**). So, we also compared the functions belonging to these three core metabolism pathways at level-3 (**Figure 8**). Lake microbial assemblages had higher abundance of KOs belonging to amino acid metabolism while stream microbial assemblages had higher abundance of KOs affiliated with carbohydrate metabolism. In stream ecosystems, OM is a heterogeneous mixture containing carbohydrates, proteins, lipids, lignins, and other compounds (Thurman, 2012). Soil and plant litter inputs as well as autochthonous material from instream primary producers contribute to OM (Webster and Meyer, 1997), which can accumulate in benthic biofilms (Ylla et al., 2011). Various heterotrophic bacteria make benthic biofilms the metabolic hotspots of OM degradation in stream ecosystems (Battin et al., 2003; McClain et al., 2003; Peter et al., 2011). In stream biofilms, carbon cycling genes were most common, followed by genes associated with other nutrient cycles (Dopheide et al., 2015). As a major form of organic nitrogen, amino acids are among the most labile fractions of bulk OM in lakes (Cowie and Hedges, 1992; Hedges et al., 1994). Their degradation not only support microbial production but enrich the biologically available pools of inorganic N forms, NO_3_, NO_2_, and NH_4_ (Stepanauskas et al., 1999). Furthermore, amino-acid-based solutes are also commonly used by bacteria for osmoregulation (Harris, 1981), which may be more important in a high salinity environment like Qinghai Lake.

For energy metabolism pathway genes, stream microbial assemblages had higher abundance of genes involved in oxidative phosphorylation and nitrogen metabolism, however, lake microbial assemblages had higher abundance of genes involved in carbon fixation and photosynthesis (**Figure 8**). In a watershed, streams play a crucial role in nitrogen metabolism, microbial nitrogen fixation, denitrification, and ammonification exert control over nitrogen exports to downstream aquatic ecosystems (Peterson et al., 2001; Valett et al., 2008). It has also been suggested that the uptake of nitrate is related to ecosystem photosynthesis, which is more intensive in lake; while denitrification is related to ecosystem respiration, which is more intensive in stream biofilms (Mulholland et al., 2008). This was in line with the physicochemical environments that lake had higher NH_4_, while stream had higher TN and NO_3_ (Supplementary Table S1). Moreover, high photosynthesis in lake microbial communities indicates more autotrophic carbon fixation in lake. However, stream biofilms have higher oxidative phosphorylation which is the most abundant and active energetic pathway (Huang et al., 2014), indicating more heterotrophic remineralization of organic carbon. This was in line with the high DOC in lake and low DOC in stream (Supplementary Table S1).

## Conclusion

In a watershed, lake and its input streams are highly connected with complex relationships. Streams receive nutrient and OM inputs from terrestrial ecosystems, subject them to internal processing, and pass them to lakes. In our study, we documented the taxonomic and functional differences between microbial communities in Qinghai Lake and its input streams. Stream biofilms and lake water harbored distinct microbial communities. The microbial communities were different taxonomically and functionally between stream and lake. Moreover, different strains of a species may have distinct functions in lake water while there might be more different taxa which have the same functional traits in stream biofilms. Stream biofilms also had a microbial network with higher connectivity and modularity than lake water. In terms of potential functions, lake microbial assemblages displayed a greater representation of many metabolism pathways while the microbial assemblages in stream biofilms were more abundant in xenobiotic biodegradation and metabolism and lipid metabolism. Furthermore, amino acid metabolism, carbon fixation, and photosynthesis were had strong representation in lake microbial assemblages while stream microbial assemblages were higher in carbohydrate metabolism, oxidative phosphorylation, and nitrogen metabolism. These results provided an understanding of stream-lake linkages from the perspective of microbial structures and functional potentials.

## Author Contributions

ZR, FW, XQ, and JE designed the study; ZR, FW, XQ, YL, and LC performed the field work and laboratory work; ZR and XQ analyzed the data; ZR and JE wrote the manuscript; all authors reviewed the manuscript.

## Conflict of Interest Statement

The authors declare that the research was conducted in the absence of any commercial or financial relationships that could be construed as a potential conflict of interest.
