# Supplementary material for: Taxonomic and Functional Differences between Microbial Communities in Qinghai Lake and Its Input Streams
Source: Front Microbiol. 2017 Nov 22;8:2319. doi: 10.3389/fmicb.2017.02319 (PMC5702853; doi:10.3389/fmicb.2017.02319)
Supplement: Supplementary file 1 [file Data_Sheet_1.docx]

# Taxonomic and functional differences between microbial communities in Qinghai Lake and its input streams

*Ze Ren^1^, Fang Wang^2,3*^, Xiaodong Qu^2,4^, James J. Elser^1^, Yang Liu^2,3^, Limin Chu^2,3^*

*^1^ Flathead Lake Biological Station, University of Montana, Polson, MT, USA*

*^2^ State Key Laboratory of Simulation and Regulation of Water Cycle in River Basin, China Institute of Water Resources and Hydropower Research, Beijing, China*

*^3^ Department of Water Resources, China Institute of Water Resources and Hydropower Research, Beijing, China*

*^4^ Department of Water Environment, China Institute of Water Resources and Hydropower Research, Beijing, China*

***Correspondence:**

Dr. Fang Wang

[wangf@iwhr.com](mailto:wangf@iwhr.com)

# Figure S1 Metagenomic functional predictions of bacterial 16S rRNA gene assemblages using PICRUSt. The relative abundance of predicted Kyoto Encyclopedia of Genes and Genomes (KEGG) orthologies (KOs) are shown at level-1. * and ** indicate KOs that were significantly different between lake and stream at the significant level P<0.05 and P<0.01, respectively.


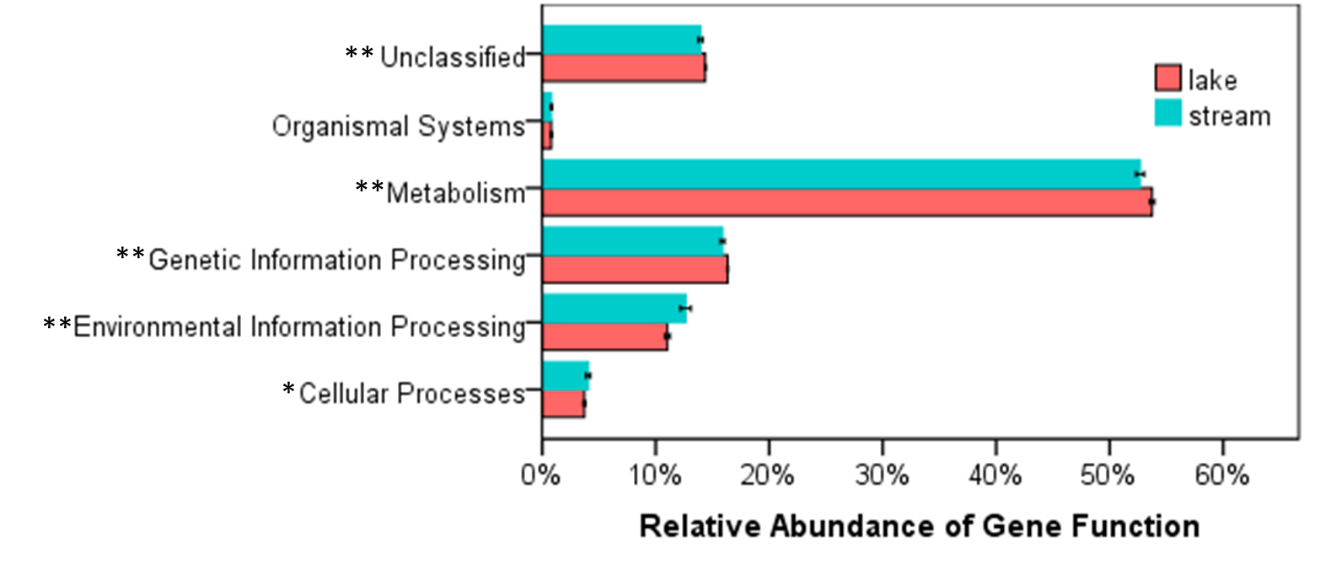


# Table S1 Basic physicochemical factors of Qinghai Lake and its input streams (Mean±SD). The factor sharing a letter is not significantly different between lake and stream, whereas the factor with different letter is significantly different (t-test, P<0.05)

|  | Lake | Stream |
| --- | --- | --- |
| Altitude | 3194±0^a^ | 3511±268^b^ |
| Temp | 14.5±1.227^a^ | 10.391±2.37^b^ |
| Cond | 15028±4220^a^ | 394±172^b^ |
| DO | 6.677±0.599^a^ | 6.369±1.539^a^ |
| pH | 8.898±0.139^a^ | 8.464±0.259^b^ |
| DOC | 10.165±0.998^a^ | 2.121±0.671^b^ |
| TN | 0.737±0.075^a^ | 1.649±1.281^b^ |
| NH4 | 0.139±0.020^a^ | 0.011±0.008^b^ |
| NO3 | 0.054±0.010^a^ | 1.483±1.246^b^ |
| TP | 0.023±0.005^a^ | 0.014±0.006^b^ |
| SRP | 0.013±0.002^a^ | 0.011±0.002^b^ |

# Table S2 Alpha diversity (Chao1, richness, Shannon index, and Simpson index) of microbial communities in Qinghai Lake and its input streams (Mean±SD) in term of OTUs and functional genes. The alpha diversity component sharing the same letter is not significantly different between lake and stream, whereas the component with different letter is significantly different (t-test, P<0.05)

|  | OTUs | | Functional Genes | |
| --- | --- | --- | --- | --- |
|  | Lake | Stream | Lake | Stream |
| Chao1 | 1029±122^a^ | 1854±331^b^ | 4764±111^a^ | 5278±301^b^ |
| Richness | 521±48^a^ | 1050±241^b^ | 4613±93^a^ | 5078±241^b^ |
| Shannon | 5.186±0.254^a^ | 7.049±0.724^b^ | 11.025±0.022^a^ | 11.070±0.066^b^ |
| Simpson | 0.922±0.023^a^ | 0.965±0.031^b^ | 0.999±0.000^a^ | 0.999±0.000^a^ |

# Table S3 Comparison of topological parameters between random and real networks using Z-test, as well as the topological parameters of two real networks (stream microbial network and lake microbial network) using T-test.

|  | Stream | | Lake | |
| --- | --- | --- | --- | --- |
|  | Random | Real | Random | Real |
| Number of Nodes | 808 | 808 | 302 | 302 |
| Network Density | 0.032 | 0.032 | 0.02 | 0.02 |
| Avg. Number of Neighbors | 26.094 | 26.094 | 5.927 | 5.927 |
| Network Centralization | 0.022±0.002* | 0.069 ^a^ | 0.027±0.004* | 0.057 ^b^ |
| Network Heterogeneity | 0.193±0.007* | 0.803 ^a^ | 0.402±0.016* | 0.739 ^b^ |
| Characteristic Path Length | 2.384±0.001* | 4.694 ^a^ | 3.401±0.011* | 4.524 ^b^ |
| Clustering Coefficient | 0.032±0.001* | 0.638 ^a^ | 0.023±0.006* | 0.304 ^b^ |
| Modularity | 0.451±0.011* | 0.955 ^a^ | 0.509±0.011* | 0.816 ^b^ |

Note: The topological parameter sharing a letter is not significantly different between the real microbial networks of lake and stream, whereas the topological parameter with different letter is significantly different (t-test, P<0.05). * indicate that for each network, the topological parameter was significantly different between the random network and the real network at the significant level P<0.05.
